# Signalling and social learning in swarms of robots

**DOI:** 10.1098/rsta.2024.0148

**Published:** 2025-01-30

**Authors:** Leo Cazenille, Maxime Toquebiau, Nicolas Lobato-Dauzier, Alessia Loi, Loona Macabre, Nathanaël Aubert-Kato, Anthony J. Genot, Nicolas Bredeche

**Affiliations:** ^1^ Universite Paris Cite, CNRS, LIED UMR 8236, Paris F-75006, France; ^2^ Sorbonne Universite, CNRS, ISIR, Paris F-75005, France; ^3^ ECE Paris, Paris, France; ^4^ Sorbonne Universite, CNRS, IBPS, Laboratoire Jean Perrin, Paris F-75005, France; ^5^ Department of Information Sciences, Ochanomizu University, Tokyo, Japan; ^6^ LIMMS (IRL2820)/CNRS-IIS, University of Tokyo, Tokyo, Japan

**Keywords:** signalling, communication, social learning, swarm robotics, decentralized learning and execution, multi-robot systems

## Abstract

This paper investigates the role of communication in improving coordination within robot swarms, focusing on a paradigm where learning and execution occur simultaneously in a decentralized manner. We highlight the role communication can play in addressing the credit assignment problem (individual contribution to the overall performance), and how it can be influenced by it. We propose a taxonomy of existing and future works on communication, focusing on information selection and physical abstraction as principal axes for classification: from low-level lossless compression with raw signal extraction and processing to high-level lossy compression with structured communication models. The paper reviews current research from evolutionary robotics, multi-agent (deep) reinforcement learning, language models and biophysics models to outline the challenges and opportunities of communication in a collective of robots that continuously learn from one another through local message exchanges, illustrating a form of social learning.

This article is part of the theme issue ‘The road forward with swarm systems’.

## 1. Introduction

A general and well-accepted definition of swarm robotics highlights the deployment of a possibly large collective of robots each with limited computation and communication capabilities working together as a result of multiple local interactions to achieve a common cause [1–6]. It is important to note that ‘limited’ does not mean ‘simple’: a hypothetical collective of idealized self-aware language-capable robots could still be considered a swarm if decentralized coordination is required owing to the inherent delay in communication, even if the environment is static. The limited capabilities of each robot are to be understood as a relative property that puts into relation two conceptual levels: (i) at the individual level, the capabilities of one individual component of the swarm, which encompass both its physical (sensors and actuators) and algorithmic (memory and computing power) capabilities and (ii) at the global level, the swarm complexity in terms of its size and spatial configuration, which define the possibilities of interactions between its components. While the hardware capabilities of the robots limit the goals that can be achieved, the limitation in software capabilities is the key factor. Whenever memory or computation is lacking at the individual level, collective action requires decentralized coordination, as each robot can only sense and act in its immediate surroundings. In addition, it is important to consider the time component of computational complexity, which depends on either or both a time-constrained task and an inherently dynamic environment. This implies that the swarm response time should be short enough for its actions to be relevant.

In this paper, we do not impose limitations over the actual capabilities of the robots or on the swarm structure (e.g. a heterogeneous swarm of unconventional robots is possible) and allow for different interpretations of what cooperation means (e.g. from just avoiding each other to displaying complex coordinated strategies). This is covered by the slightly different and more accurate definition: **swarm robotics involves deploying robotic agents that coordinate in a decentralized manner to achieve a common goal, with each robot limited to sensing and acting within its immediate environment**.^1^ This definition opens new venues for thinking about the future of the field, including bridges towards other fields with similar concerns, as we will see later.

**Figure 1.**
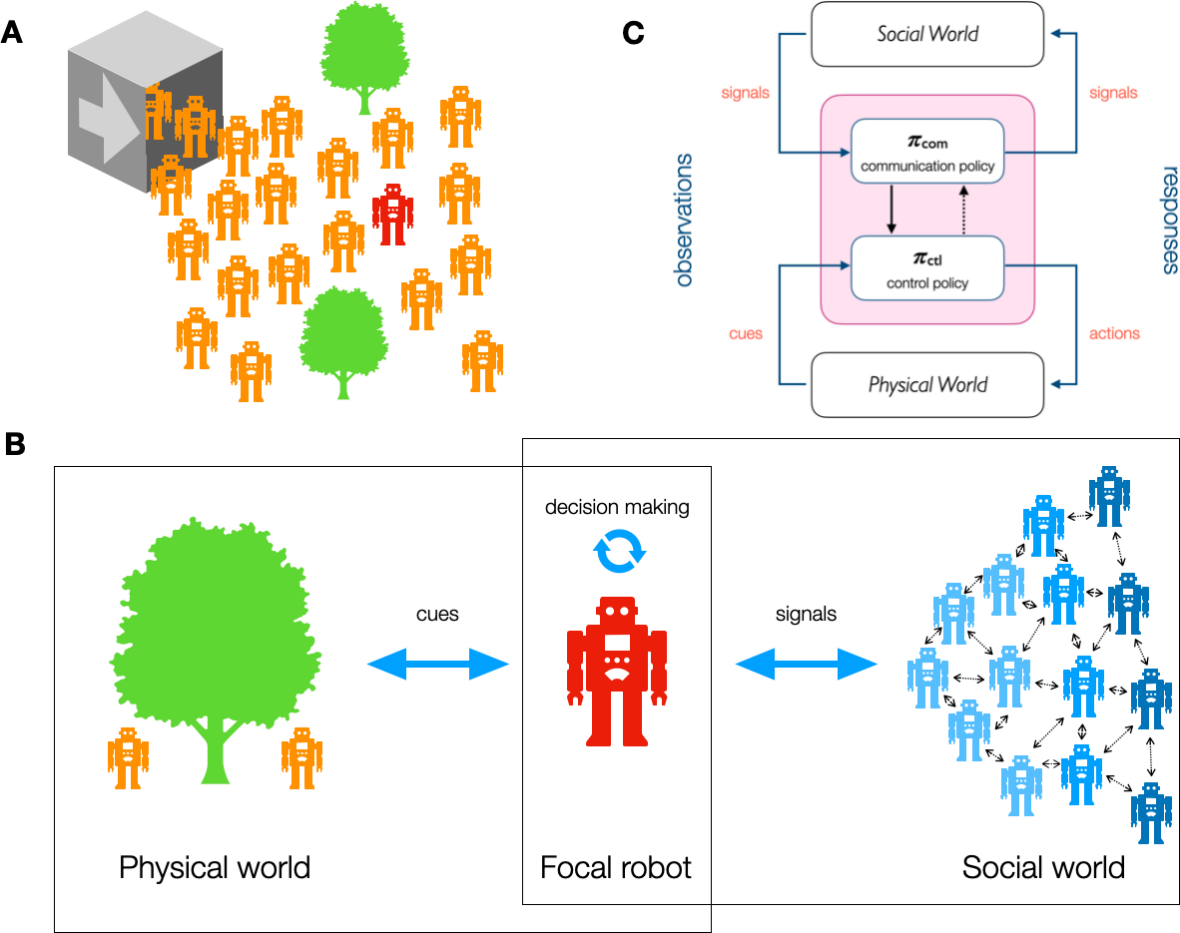
(A) A swarm of robots is deployed in an unknown environment. Robots must learn together to solve a task. Robots interact locally with nearby robots and physical elements. (B) The decision-making process of a focal robot is based on cues from the physical world and signals from the social world. (C) Diagram of the communication and control policies for a robot, distinguishing between signals for local interactions and cues from the broader environment. The pink box denotes the policy of the robot which gets information from observations (i.e. cues and signals) and produces actions (i.e. effectors and communication channels). There are two sub-policies for each process, though in practice a single general policy may be used (e.g. a single artificial neural network), or multiple policies, either ad hoc or subject to learning.

The design of efficient individual policies within a swarm of robots usually relies either on carefully crafting (possibly bio-inspired) behavioural rules or on using learning and/or evolutionary optimization algorithms. Robot policies, which are generally similar across a given swarm, do not change after deployment. While this approach is sufficient in many cases, it becomes a limitation whenever the target environment is unknown before deployment or changes over time. This is why an important effort, originally stemming from evolutionary robotics, has been made since the turn of the twenty-first century to develop decentralized online evolutionary learning algorithms. This family of algorithms aims at enabling a robot swarm to adapt continuously while already deployed in the real world, as illustrated in figure 1*a*, and has been referred to as either embodied evolutionary (EE) robotics [7] or social learning for swarm robotics [8,9]. These algorithms have achieved remarkable success in terms of the number of implementations on real robots when compared with other fields working with learning multi-robot systems (see [10] for a review).

In this paper, we posit that the class of problems addressed when using such social learning or EE algorithms is covered by the umbrella term of **decentralized learning and execution (DLE**), which designates a paradigm that will be more familiar to the wider reinforcement learning community [11,12]. This contrasts with the widely used ‘design then deploy’ paradigm, which includes (i) prior hand design, (ii) offline evolutionary robotics, and (iii) multi-agent reinforcement learning under the centralized training and decentralized execution paradigm.

We focus here on **how communication within a swarm of robots can be used to improve coordination under the DLE paradigm**. Communication can obviously be used by each robot in the swarm to enable information sharing and/or synchronized behavioural response [3,4]. However, communication can also play a role in the very nature of the learning process as all computation regarding learning under the DLE paradigm is performed in the field, without any human or central computer involved. A straight-forward form of communication in this context is exemplified by the crude control parameter sharing used in EE algorithms [7,9,13,14], where (either all or a sub-part of) the neural weights of artificial neural networks are sent from one robot to its neighbours, possibly attached with a self-assessment of its performance from local observations (details in [10]).

In §2, we start by exploring how working in the DLE paradigm raises unique challenges, whether communication among robots is enabled or not. We expose how using DLE can lead to counter-intuitive consequences owing to learning in a decentralized fashion, in particular regarding unwanted and counter-productive competition among robots. In §3, we propose a taxonomy to characterize existing and future works on communication. In §4 we offer a review of existing works that draw from several very different domains such as biophysics, evolutionary robotics, language evolution, multi-agent deep reinforcement learning and language models—to provide an overview of current and future directions. We propose a classification of communication means along the axes of information selection and physical abstraction, ranging from raw information directly available in the environment in §4a (e.g. transfer of heat or matter, either as raw information or as mathematical abstractions) up to high-level language-based communication in §4b (e.g. emergent or human-like syntax and grammar). Finally, in §5, we summarize important ideas explored earlier and provide comments and considerations for the future.

## 2. Dynamics of decentralized learning and execution

As stated in §1, natural evolution and social learning are good examples of processes working under the DLE paradigm. Individuals compete with one another to gain a selective advantage. Combined with random variations and inheritable traits, the traits of successful individuals will become more frequent over time. Of course, there is a stark contrast between natural systems and swarm robotics systems: we engineer the robot swarm to address a particular problem defined before deployment which may require coordination to be addressed (foraging, exploration, patrolling, transporting, construction or monitoring to give a few examples [15]). While the desired outcome may be relatively easy to define, the challenge is to endow each robot with the capability to assess how much it contributes to solving the task, i.e. self-assessing the robot’s contribution to the global welfare of the collective, which is itself determined by how efficiently the task is solved.

In a collective, devising the contribution of each individual is referred to as the credit assignment problem, which is well known in the multi-agent and cooperative game theory communities [16,17]. If a complete alignment of the individual’s interest with the global welfare of the collective is possible, the best actions from the viewpoint of the robot will also be the best for the collective. In a set-up where individual policies are learned, this corresponds to converging towards a Nash equilibrium that is also a social optimum, meaning none of the robots has the incentive to deviate from its current behavioural strategy as it is already the best the robot can do reward-wise (see [18–20] for theoretical considerations in distributed robotic systems, and [21] for a practical example with evolutionary learning in a swarm of robots where there is a mismatch between evolutionary stable strategies and social optimal strategies).

A direct way to make individual interests coincide with that of the team would be to provide each individual with a measure of their contribution to the global performance. However, **estimating the marginal contribution of each robot to the performance of the collective is intractable in the general case**. Even in an idealistic setting, when a scenario can be replayed an indefinite number of times and robots can be removed or added at will, computation time for estimating the marginal contributions for each individual grows exponentially with the population size as all subsets of individuals must be considered [22–24]. It is also interesting to note that the more classic reinforcement learning methods using centralized learning do not yield optimal results, as the marginal contributions of the robots are often partially or badly estimated even by the centralized critic used in multi-agent (deep) reinforcement learning [12]. One efficient simplifying hypothesis used in the field of evolutionary collective robotics is to consider a swarm of clones [25,26], turning what originally looks like a collective decision-making problem into an optimization problem as a single control parameter set is used for the entire swarm and optimized in a centralized fashion. This method is, however, not applicable under the DLE paradigm as it requires a centralized coordinator for learning.

Approximation methods to estimate on-the-fly the marginal contribution of robots in a collective exist, of course, and trade tractability against a lack of optimality or assume simplifying hypotheses on the class of problems to be addressed (see in particular [27–29]). A straightforward method is for the human supervisor to define *a priori* an explicit evaluation function embedded in each robot whose goal is to evaluate locally the performance of said robot. This is the case with most works in EE and social learning in swarm robotics, where each robot computes an estimate of its performance based solely on directly available information and self-assessment [10]. This is also the case in cooperative multi-agent learning whenever each agent is an independent learner, i.e. considering others as part of a non-stationary environment [30]. In both cases, the global performance will depend on the ability of the human engineer to design a function that provides a reliable estimate of the performance of a robot, aligning local motivation with the desired global outcome. Obviously, this can quickly become challenging as the task and/or the environment grow in complexity—e.g. foraging in a field without obstacles can be very different from foraging in a complex environment where the division of labour offers a significant advantage.

Unfortunately, **the slightest misalignment between the individual’s interests and that of the collective can lead to a suboptimal group-wise performance**. In this case, the whole swarm will eventually converge towards a Nash equilibrium that does not guarantee social optimality. This is explained by the nature of the evolutionary dynamics at work behind social learning in a swarm: elements that play a part in the behavioural strategies of the robots are competing among themselves to invade the population of robots. If the metric used to compare those elements is aligned (respectively, not aligned) with the global task, then competition will end with individual strategies that are optimal (respectively, sub-optimal) with respect to the task. This can be explained by using the famous ‘selfish gene’ metaphor popularized by Richard Dawkins [31]: robots are merely vehicles for competing units (e.g. genes or group of genes, neural network parameters, symbols from an emerging language, elements of an artificial culture, etc.) facing selective pressure.

Such evolutionary dynamics can then have a direct effect on the long-term behavioural strategies of neighbouring robots, with sometimes surprising outcomes such as mutualistic cooperation (i.e. cooperation that benefits each involved party) and altruistic behaviour (i.e. cooperation that involves a net loss at the individual level, but which indirectly benefits the survival of related individuals) [32]. In particular, **each individual’s strategy is shaped by its inclusive fitness that captures both its ability to survive *and* its ability to help related individuals** (a relation that is generally, but not always, defined at the genotypic level) [33]. Kin selection, the process by which an individual favours their relatives, is also relevant for the development of cultural adaptation [34] and language [35]. This has been shown previously to also be the case with social learning algorithms for swarm robotics [36]: robots can lose part of their survival chances to help robots with whom they share information.

**Figure 2.**
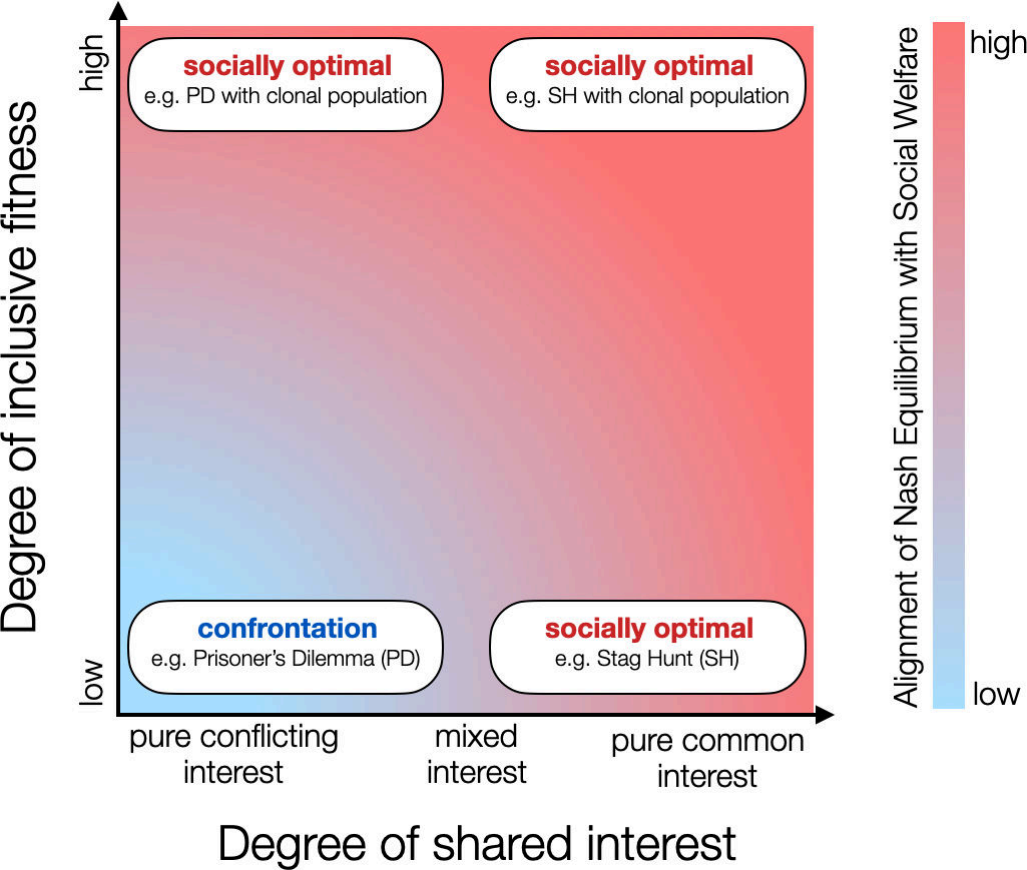
Alignment of Nash equilibrium with social welfare with respect to the degree of inclusive fitness and the degree of shared interest among robots. The x-axis shows how aligned the individual’s interest (e.g. its local fitness function) is with that of the group, which is uniquely defined by its ability to optimally solve the task. The y-axis shows the level of inclusive fitness experienced by each individual in the population (e.g. owing to kin recognition, environmental viscosity, etc.). The four text boxes on the graph provide examples using the well-known theoretical games of Prisoner’s Dilemma (a competitive game where players should defect) and Stag Hunt (a coordination game where players should cooperate) and two extremes regarding how inclusive is an individual’s fitness in a population (unrelated individuals working for their own sake versus a population of clones working for the collective).

Figure 2 combines the two concepts just discussed: (i) the stronger the alignment between the individual’s interest and the group’s welfare, the better the performance with respect to the user-defined objective (*x-*axis) and (ii) inclusive fitness, which shows the degree to which an individual’s interest is aligned with that of its relatives (*y*-axis). In this figure, we show two opposed extreme configurations, one in which individuals in the swarm are in confrontation with conflicting interests, and another one in which individuals’ interests are aligned and individuals cooperate to maximizing the social welfare, whether this incurs an individual cost or not. Obviously, the level of cooperation for solving the user-defined task will be maximal if alignment is complete, and may decrease otherwise depending on the task at hand. Much less obvious is the influence of inclusive fitness, where an individual cost may be paid for the benefit of the whole. Insight can be achieved by looking at the example of eusocial colonies (e.g. ants and termites) where the fitness of one individual is vastly defined by that of its superorganism. In this case, individual actions that benefit the group will be performed, even if they are detrimental to the individual (see [37] for a study of the effect of inclusive fitness in evolutionary collective robotics). To some extent, a high level of inclusive fitness can compensate for a misalignment between the individual’s interest and that of its conspecifics. In the figure, we formulate this relation as the degree to which the Nash equilibrium of the evolving population will converge to the socially optimal outcome with respect to the user-defined task.

We now turn our attention back to communication in a swarm of robots and the implication of previous considerations on it. Communication can be used to endow each robot with the ability to locally estimate its contribution in an online fashion, as shown by recent works in the field of cooperative multi-agent reinforcement learning that proposed using communication between robots to locally aggregate the data available on the performance of the swarm as a whole [38,39]. In this way, communication can be used to gather data on a macroscopic scale so that more information is available to each individual regarding the performance of the whole, and possibly to provide an individual’s ability to measure its contribution. Although this does not solve the credit assignment problem, communication can help to perform counterfactual reasoning to simulate hypothetical scenarios in the absence of the focal robot [40].

Unfortunately, communication also suffers from a possible misalignment between the Nash equilibrium and socially optimal strategies, especially if it evolves (e.g. emergent signalling or language). In case of misalignment, environmental contingencies and competitive pressure among individuals can lead to sub-optimal communication strategies, as evolving communication undergoes the same pressures as learning the action policy, resulting in robots developing sub-optimal communication efficiency to gain a competitive advantage against competitors [41]. In turn, evolving communication may benefit from robots with a higher degree of inclusive fitness and/or a shared interest between individuals [42].

## 3. A taxonomy of signalling methods in swarm robotics

First, let us start by narrowing the scope regarding the nature of communication we are interested in by distinguishing cues from signals. Cues provide information to the focal individual, extracted from the environment through direct observations (e.g. the relative alignment of nearby conspecifics [43,44]) or identification of body markers (e.g. a conspecific’s phenotypic trait). They do not require an identified interlocutor and, if another individual is involved, they are not produced intentionally. Signals involve an emitter and at least one receiver. They are produced intentionally by the emitter through one or several available modalities (auditory, visual, olfactory, etc.), and can vary greatly in complexity, from the production of a chemical compound to human language. The interested reader can refer to [45] for a comprehensive introduction to cues and signalling in nature.

Figure 1*b,c* provides an illustration from a robot swarm perspective. Each robot may experience both cues, observed in the physical world, and signals, originating from other robots and received through dedicated channels such as short-range proximity communication devices (e.g. infrared, visible light, radio, etc.). We explicitly limit our scope to the moment when information from the signal is readily available to the robot, leaving any pre-processing transparent (signals can be initially extracted from another modality such as speech and sign language, as is the case in robot–human communication [46]).

Communication strategies in swarm robotics cover both stigmergic communication and direct communication. Stigmergic communication works by leaving a trace in the environment [47,48], such as a virtual pheromone trail for other robots to consider [49,50]). Direct communication involves explicit exchanges of information among robots, either through pre-defined or emergent signalling strategies. In particular, emergent communication strategies evolve naturally from the interactions and the optimization processes at work within the swarm, enabling robots to converge towards efficient adaptive behaviours without centralized control.

In addition to whether signalling strategies are learned or pre-defined, the nature of the signals can vary greatly taking, for instance, discrete and continuous forms. Low-level communication methods often mimic natural processes such as diffusion, reaction and advection, enabling robots to share information about their local environment. High-level methods involve more abstract forms of communication, such as emergent or structured language models, allowing for sophisticated interactions and decision-making.

Signalling also necessarily incurs some form of restriction over the nature and the amount of information that will be shared, driven by the necessity to transfer relevant information only. This process may be lossless (e.g. suppressing redundant information, compressing information without loss or changing the way information is represented) or lossy (e.g. ignoring irrelevant information, compression with loss). In practice, as the complexity of the environment increases, so does the need for sharing only that which is relevant for the task at hand (e.g. selection attention in humans [51], or methods used to avoid the curse of dimensionality in machine learning [52]).

**Figure 3.**
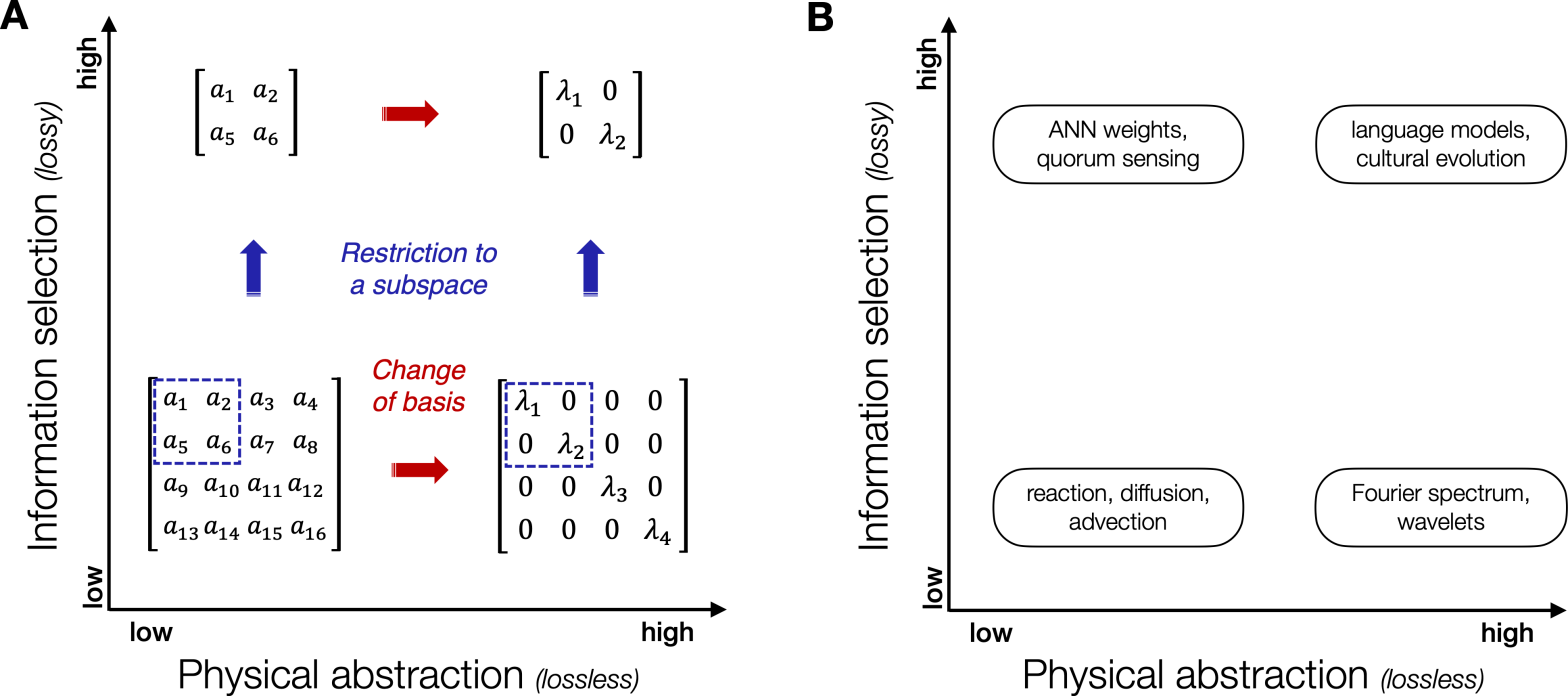
Signalling methods can be projected in a two-dimensional plane using information selection and physical abstraction as main components. (A) An algebraic analogy for information selection and physical abstraction in communication processes. Changing the degree of information selection can be done through operations like restriction to a subspace (projection with or without loss). Changing the level of physical abstraction can be done via a change of basis, such as transforming a complex matrix into a simpler diagonal form, illustrating how information can be simplified and structured. (B) ifferent approaches to communication in robotics, mapped by information selection and physical abstraction. Low-level methods include biophysics-inspired processes, while high-level methods involve language models and emergent languages.

We propose two axes for classification using the **degree of information selection** and the **degree of physical abstraction**. On one hand, information selection aims at reducing the quantity of information shared by losing information not deemed relevant. On the other hand, physical abstraction aims at changing the way information is represented without loss of information to reveal what is already present. This is illustrated in figure 3. The left-hand part of the figure provides an analogy with algebra to provide an intuition using a mathematical metaphor. The right-hand part maps well-known approaches used in swarm and collective robotics, which will be explored further in the later sections.

In the region considering a low degree of both information selection and physical abstraction, communication processes are closely tied to raw physical phenomena, such as reaction, diffusion and advection. These methods mimic natural processes to transfer information, focusing on detailed, low-level interactions. Increasing the level of physical abstraction (*x*-axis) enables the extraction of hidden but highly relevant information from raw information such as using spectrum analysis or Fourier transforms, e.g. to capture geometric information of collective spatial configurations [53]. Similarly, a second axis (*y*-axis) explores how increasing the degree of information selection can extract relevant information in different forms. For example, sharing parameters of an artificial neural network controller that maps sensory inputs to motor outputs, as is common in adaptive swarm robotics [10], can be seen as a highly compressed (and biased) instance of a reaction-diffusion process. Finally, the extremes of both axes point towards signalling strategies with high degrees of information selection and physical abstraction, where we can find, e.g. the use of a communication apparatus that is based on a large language model (LLM), enabling human-level structured perception and signalling.

## 4. Current trends in signalling for swarm robotics

In this section, we provide a review of relevant methods from swarm robotics as well as other domains, to reveal what the future states of signalling could be, considering both ad hoc and emerging signalling methods. The section follows the structure provided earlier: we first describe signalling methods with a low degree of information selection in §4a, then move up to those with a high degree of information selection in §4b. In each sub-section, strategies with different degrees of physical abstraction are described, also drawing from domains beyond that of swarm robotics. We make significant room for signalling methods used in multi-agent reinforcement learning as well as in the currently popular domain of LLMs. As mentioned in §1, we stress that while existing swarm robotics hardware is still technically limited, this state of affairs may change in the near future. As a consequence, we expect that collective systems that can be identified under the umbrella of robot swarms will feature embedded computation capabilities powerful enough to run, and possibly train in real-time, LLMs (e.g. LLMs can already run on limited hardware [54]).

### (a) Low degree of information selection

*Summary:* In this section, we investigate communication in multi-agent systems as information exchanges with minimal simplification of the baseline observable data from local agents. This involves two types of signalling schemes. Scheme (1) involves signals that reflect local observations directly. Biological examples include social insect communication and autoinducer exchanges in bacteria. In swarm robotics, these principles are applied through algorithms mimicking biological behaviours through local interaction and communication rules. Scheme (2) includes signals with a high level of physical abstraction and structure, such as the use of Fourier transforms and wavelets to analyse and share periodic patterns and multi-scale features in data. In swarm robotics, agents might share with immediate neighbours their computed gradients or neural network weights, or perform Fourier transforms or eigenspectrum analysis to understand and communicate the underlying structure of complex data.

Multi-agent communication with low information selection (lower part of figure 3) involves the direct and explicit exchange of observable information from local agents [55]. The signals are transmitted in a form that retains most of the original observations, without any extensive selection mechanism removing parts of the baseline observation data. In figure 1*c*, communication with low information selection involves minimal loss of information between the cues from the environment and their packaging into signals sent to other agents. This approach is relevant either (i) in cases where the observations already have low dimensionality, (ii) in cases where most of the observations contribute to the collective dynamics of the group or (iii) in cases where knowing in advance which parts of the observations are useful to communicate is difficult to achieve.

(**1) Low physical abstraction:** In the case with both low information selection and low physical abstraction (lower left-hand quadrant in figure 3), signals represent direct and tangible information about the environment, with minimal transformations from the observations of local agents. This type of signalling is exemplified by the following biological systems: autoinducers exchanges among bacteria [56], auditory and tactile signals in *Drosophila* [57], chemical alarms released from certain fish species to alert conspecifics of the presence of a predator [58], the bioluminescence mechanisms of fireflies for mate attraction [59], electric signals in certain fish [60] or birds songs to attract mates [61] or to signal aggressive intent [62].

Swarm robotics algorithms deployed on small robots or with self-organization capabilities also fit in this quadrant, because they rely on simple ad hoc signalling rules based directly on local states and observations, without significant loss of information or transformations. For instance, in [42] a signalling behaviour is optimized so that robots emit specific signals when they are close to an object or zone of interest. In [63], robots share all their local sensory information with their neighbours during a predator–prey task. The relative position of each robot or site of interest is locally broadcasted in [64,65]. In [66], robots can probabilistically broadcast information from one to another to assess the dynamics of information propagation.

Multi-agent systems inspired by physical dynamics can also be classified in this category: e.g. reaction-diffusion [67], chemical oscillations [68] and morphogenesis [69] can be seen as multi-agent systems where agents are spatial discretization points and global dynamics emerge from local interactions (communication without information loss). Diffusion is a fundamental physical process where particles spread from areas of higher density to areas of lower density. In multi-agent systems, diffusion can serve as a means of communication. For example, chemical signalling in cells relies on molecular diffusion to guide movement, growth and specialization. Reaction-diffusion systems involve the creation, transformation or destruction of diffusive elements through local interactions to create complex patterns. In multi-agent systems, reaction-diffusion can explain how agents interact with their environment and each other via chemical signals [70].

Moreover, making robots out of molecules allows the creation of massive swarms of millions of robots. In the last decades, researchers have used artificial DNA as computing and building blocks to develop molecular robotics [71]. Such robots can take the form of DNA origami that self-assemble into complex three-dimensional nanostructures, able to connect to each other or change configuration depending on biochemical cues [72–79]. Simpler structures can also be programmed to move on tracks [80] and sort cargoes at the nanoscale [81]. Coating beads with DNA allows to create micro-robots with higher computing capabilities, with reaction-diffusion serving to form both controllers and signals [82–84]. Another emerging field is controllable active matter, where self-propelled agents process chemical signals locally, leading to self-organization [85–91]. A final example is the Turing model of morphogenesis, which explains how patterns like animal stripes and spots emerge from the interaction of diffusing chemicals, inspiring a swarm robotics implementation where local communication mimics a reaction-diffusion system to achieve shape formation [92].

(**2) High physical abstraction:** The lower right-hand quadrant of figure 3 represents communication methods involving abstract and structured information, often detached from direct physical processes, with minimal information loss from observations.

This includes methods such as broadcasting gradients where agents locally exchange mathematical abstractions rather than direct physical signals. Gradients represent the internal state of each agent’s model, rather than a direct physical quantity. For instance, gradient propagation can compute a geodesic distance to a source robot by incrementally communicating values through neighbouring agents [93]. Gradient broadcasting can occur through microscopic rules derived from local observations [93–95], or via multi-agent reinforcement learning where the gradients of the loss function are broadcasted from agents to agents [96]. Having differentiation capabilities, i.e. access to the gradient of local states and/or messages, allows the training process to directly use this information (e.g. via gradient descent algorithms), accelerating convergence.

Eigenspectrum analysis [97] also fits this quadrant, examining eigenvalues and eigenvectors to reveal the underlying structure of data. Eigenspectrum analysis is widely used in a variety of fields, ranging from signal processing and machine learning to network analysis. This process can involve similar dynamics as those obtained in the lower left-hand quadrant—however, it will also use mathematical tools to change the representation of information without loss of information. For instance, in [53] a swarm of Kilobot robots estimates, in a decentralized way, the eigenspectrum of the communication graph between robots. Such properties are then used to reach a global consensus on the shape of the swarm, achieving arena shape recognition. This process is achieved by relying on a physics-inspired communication scheme based on the diffusion of heat across the swarm and mathematical tools to locally extract the second eigenvalue $\lambda_{2}$ of the graph Laplacian, a direct fingerprint of the arena shape containing the swarm.

Fourier transforms [98] and wavelets are other examples of abstract tools. Fourier transforms convert signals between the time and frequency domains, enabling agents to analyse and share information about periodic patterns. Wavelets decompose data into different scales, allowing agents to communicate detailed features of a signal, from broad trends to fine details. While Fourier transforms and wavelets are not yet used to process signals in swarm robotics settings, their capabilities to work with more abstract representations may allow a new class of communication schemes—e.g. to perform distributed spectral analysis as a result of communication, as in [53].

### (b) High degree of information selection

*Summary:* In this section, we explore decentralized communication as viewed from the prisms of the information bottleneck, language evolution and multi-agent reinforcement learning in situated environments. Reinforcement learning approaches to emergent communication are examined, highlighting both benefits and challenges. We emphasize the opportunities provided by LLMs for advancing communication in swarm robotics, noting their strengths in generating human-like language and reasoning, and challenges such as biases, hallucinations, embodiment and efficient deployment on robots. Overall, we present a range of approaches, with different degrees of physical abstraction, that enable decentralized agents to learn communication.

In realistic decentralized environments, all observed information is not relevant to transmit to partners. Thus, a higher degree of information selection (upper part of figure 3) is required to allow efficient transmission of the relevant information. This task can be decomposed into two complementary sub-tasks: (i) selecting relevant information and (ii) transmitting this information. The selection task involves extracting parts of the observed information that are relevant to other agents. The transmission task requires coding this information so other agents understand it while ensuring that bandwidth constraints are respected. Both tasks are highly interconnected. The selected information requires adequate means of coding to be transmitted without (or with minimal) loss. The transmission means, in turn, influence the information selection by dictating what information can be transmitted efficiently [99]. This is a form of information bottleneck, where agents need to generate a compressed mapping of their observations, that contains as much information as possible related to the task at hand [100]. As communication comes necessarily at a cost, languages operate a trade-off between meaning and compression [101,102], maximizing expressiveness while minimizing communication costs.

Studying *language games* shows how languages emerge from this information bottleneck, and from various ecological constraints. In his seminal work, Luc Steels [103] demonstrated that having a dynamic population of embodied agents, whose reasoning is unknown to one another, motivates the emergence of a shared compositional language. In the iterated learning framework [104,105], the emphasis is put on a transmission bottleneck that occurs when language is transmitted between successive generations of agents, driving languages to adopt simple and compositional structures. Later works have shown that emergent languages are also shaped by environmental [106,107] and physiological [108] constraints. These experiments highlight the different requirements for languages to originate in populations of independent agents, and demonstrate the emergence of efficient naming and grammatical conventions [102,109–111].

However, these language games still heavily simplify the context of communication interactions, by making the agents, their observations, and their actions, solely defined by the communication game. Previous works have classified this kind of setting as *non-situated* [112], as opposed to *situated* agents that have a localized existence and can physically interact with their surroundings. In situated environments, communication is one of many interfacing processes. It can be used for communicating not only about observations, but also about intents, or even about task-agnostic and abstract concepts. It may involve non-cooperative agents. It might not even be required at all times. In such realistic settings, choosing which information is relevant to communicate is a much more complex task that involves reasoning about the current state of the environment, the agent’s objective and the current knowledge and reasoning of other agents. In that sense, learning to communicate is inherently a multi-agent problem of learning how to behave in a dynamic, partially observable environment.

Recently, research in multi-agent reinforcement learning has tackled such situated environments, where performance depends on a combination of physical and communication behaviour [113]. In this context, multi-agent systems learn, often with centralized training and decentralized execution, to generate messages that participate in maximizing future returns. Here, messages are continuous vectors generated by neural networks inside the agents’ system. This makes communication a *differentiable sub-step of the action selection process*, which can be learned fully end-to-end as a tool for maximizing returns [114–117]. Because the message generation is differentiable, gradients can flow between agents. Thus, messages are explicitly trained to help other agents maximize their rewards. This approach has been extended in various ways for more targeted information sharing [118–120] or to limit bandwidth usage [121–124]. Similar approaches have been developed using discrete symbols for communication [125–129]. In those, agents have to reach a consensus on the meaning of each symbol through trial and error. The compression constraint depends both on the size of the vocabulary and the size of the sequences. Previous works have shown that imposing constraints on both of these attributes induces emergent languages to develop common characteristics of natural languages such as compositionality [130,131] and abbreviation of frequent words [132].

However, learning emergent communication through this task-oriented reinforcement learning process has many important limitations. As already mentioned, it often requires a centralized learning algorithm to allow reinforcement learning tools to reliably converge to adequate solutions. As with all gradient-based learning methods, it acts as a black box that lacks practical ways of interpreting and measuring its efficiency [133,134]. More importantly, differentiable emergent communication gives no guarantee of learning to communicate about concepts from the environment. Rather, guided by return maximization, agents converge to a consensus that may seem random to the human eye [135]. This lacks ways of anchoring its concepts in environmental, task-agnostic modalities. This is akin to the problem of *symbol grounding* [136]. Having emergent communication grounded in meanings from the environment would allow decentralized agents to learn to communicate about concepts that are shared with other agents, making learning easier and communication more efficient [103,106,137].

Following this idea, *grounding* approaches have been explored in multi-agent reinforcement learning. By linking communication with visual data [138,139], natural language [140–143] or both [144,145], agents learn to generate messages using task-agnostic concepts. In other words, they learn to use concepts dictated by external modalities to transmit information efficiently, instead of searching for a consensus on their own, starting from scratch and guided only by rewards. Agents may acquire ‘grounded’ knowledge through a variety of techniques: pre-training on a supervised task [140,144–146], alternating between supervision and self-play [146,147], optimizing the supervised learning and reinforcement learning objective at the same time [139,142,143,145,148], or constructing additional rewards based on supervised models [144]. The nature of the subsidiary tasks depends on the desired type of grounding. An autoencoding task can be added to ensure agents communicate about their observations [139,148]. To ground communication in natural language, agents can be shown examples of human-generated sentences [138,140,142,143] or learn to generate similar outputs as pre-trained language models [141,145]. A challenge when learning to use natural language is to avoid language drift [145], requiring constant supervision to prevent agents from forgetting the intended use of the given language [144–146]. Natural language offers an efficient solution to the information bottleneck problem while allowing effortless interpretation and teaming with unknown agents (human or artificial).

When using natural language, an obvious solution is to turn to LLMs. In addition to being extremely good for generating human-like sentences, they can also be grounded in visual and behavioural modalities [149]. Their context window can be exploited in various ways to insert factual information or state objectives to achieve and particular behaviours to adopt [150]. This is thanks to two important aspects of training the LLMs. First, the language-modelling pre-training phase shows the model of how humans formulate their reasoning in natural language. Second, the explicit instruction-following task optimized with reinforcement learning from human feedback [151] trains the LLM to pay close attention to what has been requested and how it should be answered. Consequently, LLMs can be used as a basis for modelling interacting agents [149,150,152]. Such *agent-based* LLMs are given information about the environment, the task, their identity and their role in the environment, all inside an initial prompt. Following this initialization, they observe and act in the environment through visual, textual and physical inputs and outputs [150,152]. Thanks to their reasoning and conversing skills, LLM agents can discuss their knowledge and intents with partners before selecting an action [153]. This can be pushed even further with personas assigned to each LLM agent, allowing a large diversity of different behaviours and offering the advantages of collective reasoning [154–158].

LLMs offer a nice playground for multi-agent interactions. They efficiently emulate human reasoning and communication. Their built-in interactivity provides a great tool for interpretation [159] and human–agent interactions [153,160,161]. However, several issues with LLMs remain and need addressing. First, embodying LLMs is a challenge requiring links to be made between language and environmental modalities (visual and behavioural). The current development of multi-modal LLMs is a step towards solving this challenge [149]. But, these approaches often require a costly fine-tuning phase to adapt the model to its new modalities. A subsequent problem is the deployment of LLM-based agents on small robotic platforms, which requires engineering work to adapt to the constraints of such platforms. This is especially true for decentralized robots that must be self-sufficient and are often limited in memory and computing power. Furthermore, we need ways of countering the intrinsic biases present in human-generated data that LLMs inevitably reproduce [162]. Last, the problem of hallucinations remains an important obstacle. LLMs are known for inventing information and being reluctant to admit when they are wrong [163]. This can lead to issues ranging from deception to breaking the simulation, which requires more work on methods for detecting, measuring and avoiding these hallucinations. While these issues can, and will certainly be addressed, this reminds us that other solutions using smaller models also work and might be preferable in many situations.

To conclude, we see that many approaches exist for teaching decentralized agents to communicate about high-dimensional environmental features. They rely on languages that select information to communicate more efficiently. These languages abstract physical elements of the world by grounding symbols in environmental features, allowing the establishment of conventions on how information should be transmitted. Different degrees of physical abstraction may serve different purposes. A group of agents specialized in a single task may be content with low physically abstracted differentiable emergent communication learned from task reward. On the other hand, established concepts and grammatical rules provide the tools to generalize acquired knowledge, compose new ideas from fundamental language blocks and communicate with unknown partners. Thus, higher physical abstraction, found in natural languages, is better suited to handle more general settings.

## 5. Conclusion

We have explored how communication through signalling can be crucial in enhancing coordination within robot swarms operating under the DLE paradigm. We have proposed a structured framework to classify existing and future signalling methods, covering a wide range of information selection levels and physical abstractions. Throughout the paper, we advocate that swarm robotics with distributed online learning capabilities offer unique challenges, for which communication can play a positive role, but to which communication is also subject. The key messages of our paper are summarized hereafter.

### (a) A path towards complex communication strategies

Earlier works in swarm robotics were closely inspired by social insects. The current state-of-the-art in swarm robotics now shows a great variety of applications and robotics set-ups, including dense to sparse swarms with homogeneous or heterogeneous robots. To account for the fast-paced advances in hardware and software, it is important to keep in mind that swarm robotics is about the relation between microscopic interactions and macroscopic organization, which remains valid even if powerful computation and signalling capabilities are available. A practical consequence we envision is the advent of robots using LLMs, composing a society of embodied agents with human-like signalling capabilities that are still bound by environmental contingencies (e.g. local communication only, complex physical interactions). Beyond the anticipated gains in performance, incorporating human language-like capabilities can offer valuable benefits with respect to explainability and human–robot interaction through the use of a shared language.

### (b) Decentralized learning and adaptive dynamics in swarm robotics present a unique challenge

Addressing the problem of distributed credit assignment is a well-known challenge in multi-agent systems. However, conducting learning in a decentralized and online fashion adds another layer of complexity, especially when policy parameters hop from one robot to the next. A consequence is that nearby robots can share similar parameters, which can indirectly cause either altruistic or competitive behaviours depending on the degree of relation between individuals (see §2). Differing from natural systems where the population may grow, the fixed size of a robot swarm affects where competition occurs: robots are mere resources for which policy parameters are competing, rather than the opposite. Exploring the long-term adaptive dynamics of behavioural strategies (in which signalling is included) in dynamic and unpredictable environments will be critical for developing adaptive and resilient swarm systems. This opens up an exciting avenue, requiring an interdisciplinary research effort, integrating expertise from fields such as evolutionary game theory [164], collective decision-making [16], evolutionary dynamics [165], sociophysics [166,167], physics of active matter [168], evolutionary computation [169] and machine learning [170].

We conclude with a list of take-home messages, targeting the three communities we believe will be at the centre of this coming revolution:

— **Researchers in swarm robotics**: simple robots are not inherently ‘simple’. What matters is the emergence of complex behaviours from microscopic interactions. Whether you work with large or small robots, dense or sparse populations, or few or many robots, all are welcome under the broad aim of continual learning in swarm systems.
— **Researchers in machine learning**: this is all about embodiment. Swarm robotics introduces a unique category of machine learning problems with elements of ‘social’ learning across physically embodied agents. Anchoring language models in physical systems brings new challenges and capabilities in distributed, online learning.
— **Researchers in complex systems**: swarm robotics provides a controllable model for exploring active matter, sociophysics models, reaction-diffusion and diffusion biophysics processes. Swarm robotics offers an experimental platform for addressing fundamental questions about adaptive collective systems.

We believe that DLE will inevitably become more prominent in swarm robotics, with signalling playing a fundamental role. We intend for this paper to serve as a milestone in shaping the future of this field by providing a framework to understand the complexities and potentials of swarm robotics, where local interactions drive continuously learning embodied agents equipped with complex signalling mechanisms.

## Data Availability

This article has no additional data.

## Footnotes

^1^
We consider swarms of self-interested robotic agents as off-topic from the present study.

## References

[1] Beni G, Wang J. 1993 Swarm Intelligence in Cellular Robotic Systems. In NATO ASI. (10.1007/978-3-642-58069-7_38)

[2] Dudek G, Jenkin M, Milios E, Wilkes D. 1993 A taxonomy for swarm robots. In 1993 IEEE/RSJ International Conference on Intelligent Robots and Systems (IROS ’93), Yokohama, Japan, vol. 1, pp. 441–447, (10.1109/IROS.1993.583135)

[3] Brambilla M, Ferrante E, Birattari M, Dorigo M. 2013 Swarm robotics: a review from the swarm engineering perspective. Swarm Intell. **7**, 1–41. (10.1007/s11721-012-0075-2)

[4] Hamann H. 2018 Introduction to Swarm Robotics. In Swarm robotics: a formal approach, pp. 1–32. Springer. (10.1007/978-3-319-74528-2_1)

[5] Dorigo M, Theraulaz G, Trianni V. 2020 Reflections on the future of swarm robotics. Sci. Robot. **5**. (10.1126/scirobotics.abe4385) 33298518

[6] Floreano D, Lipson H. 2021 From individual robots to robot societies. Sci. Robot. **6**. (10.1126/scirobotics.abk2787) 34321349

[7] Watson RA, Ficici SG, Pollack JB. 2002 Embodied Evolution: Distributing an evolutionary algorithm in a population of robots. Robot. Auton. Syst. **39**, 1–18. (10.1016/s0921-8890(02)00170-7)

[8] Heinerman J, Rango M, Eiben AE. 2015 Evolution, Individual Learning, and Social Learning in a Swarm of Real Robots. In 2015 IEEE Symposium Series on Computational Intelligence (SSCI), Cape Town, South Africa, pp. 1055–1062. IEEE. (10.1109/SSCI.2015.152)

[9] Bredeche N, Fontbonne N. 2022 Social learning in swarm robotics. Phil. Trans. R. Soc. B **377**, 20200309. (10.1098/rstb.2020.0309) 34894730 PMC8666954

[10] Bredeche N, Haasdijk E, Prieto A. 2018 Embodied Evolution in Collective Robotics: A Review. Front. Robot. AI **5**. (10.3389/frobt.2018.00012) PMC7806005 33500899

[11] Zhang K, Yang Z, Liu H, Zhang T, Basar T. 2018 Fully decentralized multi-agent reinforcement learning with networked agents. In International Conference on Machine Learning, pp. 5872–5881. (10.1109/CDC.2018.8619581)

[12] Lyu X, Xiao Y, Daley B, Amato C. 2021 Contrasting centralized and decentralized critics in multi-agent reinforcement learning. arXiv.

[13] Ficici SG, A.Watson R, B.Pollack J. 1999 ‘Embodied evolution. In a response to challenges in evolutionary robotics.’Proceedings of the Eighth European Workshop on Learning Robots, pp. 14–22. Citeseer.

[14] Bredeche N, Montanier JM. 2010 Environment-Driven Embodied Evolution in a Population of Autonomous Agents. In International conference on parallel problem solving from nature, pp. 290–299. Springer. (10.1007/978-3-642-15871-1_30)

[15] Schranz M, Umlauft M, Sende M, Elmenreich W. 2020 Swarm Robotic Behaviors and Current Applications. Front. Robot. AI **7**, 36. (10.3389/frobt.2020.00036) 33501204 PMC7805972

[16] Nisan N, Roughgarden T, Tardos É, Vazirani VV. 2007 Algorithmic game theory, pp. xvii–xviii. New York: Cambridge University Press. (10.1017/cbo9780511800481.002)

[17] Oroojlooy A, Hajinezhad D. 2023 A review of cooperative multi-agent deep reinforcement learning. Appl. Intell. **53**, 13677–13722. (10.1007/s10489-022-04105-y)

[18] Wolpert DH, Tumer K. 1999 An introduction to collective intelligence. arXiv.

[19] Stone P, Kaminka G, Kraus S, Rosenschein J. 2010 Ad Hoc Autonomous Agent Teams: Collaboration without Pre-Coordination. In Proceedings of the AAAI conference on Artificial intelligence, vol. **24**, pp. 1504–1509, (10.1609/aaai.v24i1.7529)

[20] Marden JR, Shamma JS. 2018 Game Theory and Control. Annu. Rev. Control Robot. Auton. Syst. **1**, 105–134. (10.1146/annurev-control-060117-105102)

[21] Ecoffet P, Bredeche N, André JB. 2021 Nothing better to do? Environment quality and the evolution of cooperation by partner choice. J. Theor. Biol. **527**, 110805. (10.1016/j.jtbi.2021.110805) 34107279

[22] Shapley LS. 1953 A Value for n-Person Games. In Annals of Mathematical Studies, pp. 307–318, vol. 28. Princeton University Press. (10.1515/9781400881970-018)

[23] Shoham Y, Leyton-Brown K. 2008 Multiagent systems: algorithmic, game-theoretic, and logical foundations. Cambridge University Press. (10.1017/CBO9780511811654)

[24] Wooldridge M. 2009 An introduction to multiagent systems. John wiley & sons.

[25] Trianni V, Dorigo M. 2006 Self-organisation and communication in groups of simulated and physical robots. Biol. Cybern. **95**, 213–231. (10.1007/s00422-006-0080-x) 16821036

[26] Waibel M, Keller L, Floreano D. 2009 Genetic Team Composition and Level of Selection in the Evolution of Cooperation. IEEE Trans. Evol. Comput. **13**, 648–660. (10.1109/tevc.2008.2011741)

[27] Wolpert D, Tumer K Swanson K 2000 Optimal wonderful life utility functions in multi-agent systems

[28] Kolpaczki P, Bengs V, Muschalik M, Hüllermeier E. 2024 Approximating the Shapley Value without Marginal Contributions. In Proceedings of the AAAI conference on Artificial Intelligence, vol. **38**, pp. 13246–13255, (10.1609/aaai.v38i12.29225)

[29] Wang J, Hong Y, Wang J, Xu J, Tang Y, Han QL, Kurths J. 2022 Cooperative and Competitive Multi-Agent Systems: From Optimization to Games. IEEE/CAA J. Autom. Sin. **9**, 763–783. (10.1109/jas.2022.105506)

[30] Gronauer S, Diepold K. 2022 Multi-agent deep reinforcement learning: a survey. Artif. Intell. Rev. **55**, 895–943. (10.1007/s10462-021-09996-w)

[31] Dawkins R. 2016 The selfish gene. Oxford University press.

[32] West SA, Griffin AS, Gardner A. 2007 Social semantics: altruism, cooperation, mutualism, strong reciprocity and group selection. J. Evol. Biol. **20**, 415–432. (10.1111/j.1420-9101.2006.01258.x) 17305808

[33] Hamilton WD. 1964 The genetical evolution of social behaviour. II. J. Theor. Biol. **7**, 17–52. (10.1016/0022-5193(64)90039-6) 5875340

[34] Richerson PJ, Boyd R. 2008 Not by genes alone: how culture transformed human evolution. University of Chicago press.

[35] Smith EA. 2010 Communication and collective action: language and the evolution of human cooperation. Evol. Hum. Behav. **31**, 231–245. (10.1016/j.evolhumbehav.2010.03.001)

[36] Montanier JM, Bredeche N. 2011 Surviving the tragedy of commons emergence of altruism in a population of evolving autonomous agents. In European Conference on Artificial Life.

[37] Waibel M, Floreano D, Keller L. 2011 A Quantitative Test of Hamilton’s Rule for the Evolution of Altruism. PLoS Biol. **9**, e1000615. (10.1371/journal.pbio.1000615) 21559320 PMC3086867

[38] Zhang K, Yang Z, Başar T. 2021 Decentralized multi-agent reinforcement learning with networked agents: recent advances. Front. Inf. Technol. Electron. Eng. **22**, 802–814. (10.1631/fitee.1900661)

[39] Zimmer M, Glanois C, Siddique U, Weng P. 2021 Learning fair policies in decentralized cooperative multi-agent reinforcement learning. In International Conference on Machine Learning, pp. 12967–12978.

[40] Foerster J, Farquhar G, Afouras T, Nardelli N, Whiteson S. 2018 Counterfactual Multi-Agent Policy Gradients. In Proceedings of the AAAI conference on artificial intelligence. vol. **32**. (10.1609/aaai.v32i1.11794)

[41] Wischmann S, Floreano D, Keller L. 2012 Historical contingency affects signaling strategies and competitive abilities in evolving populations of simulated robots. Proc. Natl Acad. Sci. USA **109**, 864–868. (10.1073/pnas.1104267109) 22215591 PMC3271901

[42] Floreano D, Mitri S, Magnenat S, Keller L. 2007 Evolutionary Conditions for the Emergence of Communication in Robots. Curr. Biol. **17**, 514–519. (10.1016/j.cub.2007.01.058) 17320390

[43] Reynolds CW. 1987 Flocks, herds and schools: A distributed behavioral model. ACM SIGGRAPH Comput. Graph. **21**, 25–34. (10.1145/37402.37406)

[44] Vicsek T, Czirók A, Ben-Jacob E, Cohen I, Shochet O. 1995 Novel Type of Phase Transition in a System of Self-Driven Particles. Phys. Rev. Lett. **75**, 1226–1229. (10.1103/PhysRevLett.75.1226) 10060237

[45] Smith JM, Harper D. 2003 Animal signals. Oxford University Press. (10.1093/oso/9780198526841.001.0001)

[46] van den Berghe R, Verhagen J, Oudgenoeg-Paz O, van der Ven S, Leseman P. 2019 Social Robots for Language Learning: A Review. Rev. Educ. Res. **89**, 259–295. (10.3102/0034654318821286)

[47] Bonabeau E, Dorigo M, Theraulaz G. 2000 Inspiration for optimization from social insect behaviour. Nature **406**, 39–42. (10.1038/35017500) 10894532

[48] Detrain C, Deneubourg JL. 2008 Collective Decision-Making and Foraging Patterns in Ants and Honeybees. In Advances in insect physiology, pp. 123–173, vol. 35. Elsevier. (10.1016/s0065-2806(08)00002-7)

[49] Bonabeau E, Dorigo M, Theraulaz G. 1999 Swarm intelligence: from natural to artificial systems. Oxford university press. (10.1093/oso/9780195131581.001.0001)

[50] Campo A, Gutiérrez A, Nouyan S, Pinciroli C, Longchamp V, Garnier S, Dorigo M. 2010 Artificial pheromone for path selection by a foraging swarm of robots. Biol. Cybern. **103**, 339–352. (10.1007/s00422-010-0402-x) 20644952

[51] Carrasco M. 2011 Visual attention: The past 25 years. Vis. Res. **51**, 1484–1525. (10.1016/j.visres.2011.04.012) 21549742 PMC3390154

[52] Bellman R. 1966 Dynamic programming. Science **153**, 34–37. (10.1126/science.153.3731.34) 17730601

[53] Cazenille L, Lobato-Dauzier N, Loi A, Ito M, Marchal O, Aubert-Kato N, Bredeche N, Genot A. 2024 Hearing the Shape of an Arena with Spectral Swarm Robotics. arXiv.

[54] Gerganov G. 2023 llama.cpp. See https://github.com/ggerganov/llama.cpp (accessed 30 May 2024).

[55] Ji P *et al*. 2023 Signal propagation in complex networks. Phys. Rep. **1017**, 1–96. (10.1016/j.physrep.2023.03.005)

[56] Taga ME, Bassler BL. 2003 Chemical communication among bacteria. Proc. Natl Acad. Sci. USA **100**, 14549–14554. (10.1073/pnas.1934514100) 12949263 PMC304117

[57] LaRue KM, Clemens J, Berman GJ, Murthy M. 2015 Acoustic duetting in Drosophila virilis relies on the integration of auditory and tactile signals. eLife **4**, e07277. (10.7554/eLife.07277) 26046297 PMC4456510

[58] Bairos‐Novak KR, Ferrari MCO, Chivers DP. 2019 A novel alarm signal in aquatic prey: Familiar minnows coordinate group defences against predators through chemical disturbance cues. J. Anim. Ecol. **88**, 1281–1290. (10.1111/1365-2656.12986) 30997683

[59] Marques SM, Esteves da Silva JCG. 2009 Firefly bioluminescence: A mechanistic approach of luciferase catalyzed reactions. IUBMB Life **61**, 6–17. (10.1002/iub.134) 18949818

[60] Hopkins CD. Eelectric communication in fish. Am. Sci. **62**, 426–437. 4851599

[61] Eriksson D, Wallin L. 1986 Male bird song attracts females ? a field experiment. Behav. Ecol. Sociobiol. **19**, 297–299. (10.1007/bf00300645)

[62] Searcy WA, Anderson RC, Nowicki S. 2006 Bird song as a signal of aggressive intent. Behav. Ecol. Sociobiol. **60**, 234–241. (10.1007/s00265-006-0161-9)

[63] Rodrigues T, Duarte M, Figueiró M, Costa V, Oliveira SM, Christensen AL. 2015 Overcoming Limited Onboard Sensing in Swarm Robotics Through Local Communication. In Transactions on computational collective intelligence XX, pp. 201–223. Springer. (10.1007/978-3-319-27543-7_10)

[64] Talamali MS, Saha A, Marshall JAR, Reina A. 2021 When less is more: Robot swarms adapt better to changes with constrained communication. Sci. Robot. **6**, f1416. (10.1126/scirobotics.abf1416) 34321345

[65] McGuire KN, De Wagter C, Tuyls K, Kappen HJ, de Croon GCHE. 2019 Minimal navigation solution for a swarm of tiny flying robots to explore an unknown environment. Sci. Robot. **4**, w9710. (10.1126/scirobotics.aaw9710) 33137730

[66] Hafnaoui I, Nicolescu G, Beltrame G. 2019 Timing Information Propagation in Interactive Networks. Sci. Rep. **9**, 4442. (10.1038/s41598-019-40801-5) 30872733 PMC6418309

[67] Crank J. 1979 The mathematics of diffusion. Oxford university press.

[68] Shanks N. 2001 Modeling Biological Systems: The Belousov-Zhabotinsky reaction. Found. Chem. **3**, 33–53.

[69] TURING A. 1990 The chemical basis of morphogenesis. Bull. Math. Biol. **52**, 153–197. (10.1016/s0092-8240(05)80008-4) 2185858

[70] Arai T, Yoshida E, Ota J. Information diffusion by local communication of multiple mobile robots. In IEEE Systems Man and Cybernetics Conference - SMC, Le Touquet, France, vol. 4, pp. 535–540, IEEE. (10.1109/ICSMC.1993.390769)

[71] Murata S. 2022 Introduction: Welcome to Molecular Robotics! In Molecular robotics, pp. 1–11. Springer. (10.1007/978-981-19-3987-7_1)

[72] Nummelin S, Shen B, Piskunen P, Liu Q, Kostiainen MA, Linko V. 2020 Robotic DNA Nanostructures. ACS Synth. Biol. **9**, 1923–1940. (10.1021/acssynbio.0c00235) 32589832 PMC7467825

[73] Douglas SM, Bachelet I, Church GM. 2012 A Logic-Gated Nanorobot for Targeted Transport of Molecular Payloads. Science **335**, 831–834. (10.1126/science.1214081) 22344439

[74] Torelli E, Marini M, Palmano S, Piantanida L, Polano C, Scarpellini A, Lazzarino M, Firrao G. 2014 A DNA Origami Nanorobot Controlled by Nucleic Acid Hybridization. Small **10**, 2918–2926. (10.1002/smll.201400245) 24648163

[75] Kuzuya A, Ohya Y. 2014 Nanomechanical Molecular Devices made of DNA Origami. Accounts Chem. Res. **47**, 1742–1749. (10.1021/ar400328v) 24772996

[76] Amir Y, Abu-Horowitz A, Bachelet I. 2015 Folding and Characterization of a Bio-responsive Robot from DNA Origami. J. Vis. Exp. e51272. (10.3791/51272-v) 26709748 PMC4692774

[77] Kaminka GA, Spokoini-Stern R, Amir Y, Agmon N, Bachelet I. 2017 Molecular Robots Obeying Asimov’s Three Laws of Robotics. Artif. Life **23**, 343–350. (10.1162/artl_a_00235) 28786728

[78] Daljit Singh JK, Luu MT, Abbas A, Wickham SFJ. 2018 Switchable DNA-origami nanostructures that respond to their environment and their applications. Biophys. Rev. **10**, 1283–1293. (10.1007/s12551-018-0462-z) 30280371 PMC6233340

[79] Li S *et al*. 2018 A DNA nanorobot functions as a cancer therapeutic in response to a molecular trigger in vivo. Nat. Biotechnol. **36**, 258–264. (10.1038/nbt.4071) 29431737

[80] Wickham SFJ, Bath J, Katsuda Y, Endo M, Hidaka K, Sugiyama H, Turberfield AJ. 2012 A DNA-based molecular motor that can navigate a network of tracks. Nat. Nanotechnol. **7**, 169–173. (10.1038/nnano.2011.253) 22266636

[81] Thubagere AJ *et al*. 2017 A cargo-sorting DNA robot. Science **357**, n6558. (10.1126/science.aan6558) 28912216

[82] Gines G, Zadorin AS, Galas JC, Fujii T, Estevez-Torres A, Rondelez Y. 2017 Microscopic agents programmed by DNA circuits. Nat. Nanotechnol. **12**, 351–359. (10.1038/nnano.2016.299) 28135261

[83] Aubert-Kato N *et al*. 2017 Evolutionary optimization of self-assembly in a swarm of bio-micro-robots. In Proceedings of the Genetic and Evolutionary Computation Conference, Berlin Germany, pp. 59–66. New York, NY, USA. (10.1145/3071178.3071289). https://dl.acm.org/doi/proceedings/10.1145/3071178.

[84] Cazenille L, Bredeche N, Aubert-Kato N. 2019 Exploring Self-Assembling Behaviors in a Swarm of Bio-micro-robots using Surrogate-Assisted MAP-Elites. In IEEE Symposium Series on Computational Intelligence (SSCI), Xiamen, China, pp. 238–246. IEEE. (10.1109/SSCI44817.2019.9003047). https://ieeexplore.ieee.org/xpl/mostRecentIssue.jsp?punumber=8975711.

[85] Ziepke A, Maryshev I, Aranson IS, Frey E. 2022 Multi-scale organization in communicating active matter. Nat. Commun. **13**, 6727. (10.1038/s41467-022-34484-2) 36344567 PMC9640622

[86] Wang Y, Chen H, Xie L, Liu J, Zhang L, Yu J. 2024 Swarm Autonomy: From Agent Functionalization to Machine Intelligence. Adv. Mater. e2312956. (10.1002/adma.202312956) 38653192 PMC11733729

[87] Grauer J, Jan Schwarzendahl F, Löwen H, Liebchen B. 2024 Optimizing collective behavior of communicating active particles with machine learning. Mach. Learn. **5**, 015014. (10.1088/2632-2153/ad1c33)

[88] Lavergne FA, Wendehenne H, Bäuerle T, Bechinger C. 2019 Group formation and cohesion of active particles with visual perception–dependent motility. Science **364**, 70–74. (10.1126/science.aau5347) 30948548

[89] Keya JJ, Suzuki R, Kabir AMdR, Inoue D, Asanuma H, Sada K, Hess H, Kuzuya A, Kakugo A. 2018 DNA-assisted swarm control in a biomolecular motor system. Nat. Commun. **9**, 453. (10.1038/s41467-017-02778-5) 29386522 PMC5792447

[90] Akter M *et al*. 2022 Cooperative cargo transportation by a swarm of molecular machines. Sci. Robot. **7**, m0677. (10.1126/scirobotics.abm0677) 35442703

[91] Aubert-Kato N, Nitschke G, Kawamata I, Kakugo A. 2023 Collective Cargo Transport and Sorting with Molecular Swarms. In Artificial Life Conference Proceedings, One Rogers Street, Cambridge. MIT Press. (10.1162/isal_a_00593). https://direct.mit.edu/isal/isal/volume/35.

[92] Slavkov I, Carrillo-Zapata D, Carranza N, Diego X, Jansson F, Kaandorp J, Hauert S, Sharpe J. 2018 Morphogenesis in robot swarms. Sci. Robot. **3**. (10.1126/scirobotics.aau9178) 33141694

[93] Rubenstein M, Cornejo A, Nagpal R. 2014 Programmable self-assembly in a thousand-robot swarm. Science **345**, 795–799. (10.1126/science.1254295) 25124435

[94] Gauci M, Ortiz M, Rubenstein M, Nagpal R. 2017 ‘Error cascades in collective behavior: a case study of the gradient algorithm on 1000 physical agents. In ’Proceedings of the 16th Conference on Autonomous Agents and MultiAgent Systems, pp. 1404–1412.

[95] Wang H, Rubenstein M. 2020 A Fast, Accurate, and Scalable Probabilistic Sample-Based Approach for Counting Swarm Size. In IEEE International Conference on Robotics and Automation (ICRA), Paris, France, pp. 7180–7185. IEEE. (10.1109/ICRA40945.2020.9196529). https://ieeexplore.ieee.org/xpl/mostRecentIssue.jsp?punumber=9187508.

[96] Busoniu L, Babuska R, De Schutter B. 2008 A Comprehensive Survey of Multiagent Reinforcement Learning. IEEE Trans. Syst. Man Cybern. Part C **38**, 156–172. (10.1109/tsmcc.2007.913919)

[97] Saad Y. 2011 Numerical methods for large eigenvalue problems. SIAM. (10.1137/1.9781611970739). See http://epubs.siam.org/doi/book/10.1137/1.9781611970739.

[98] Alllan VO. 1999 Discrete-time signal processing. pearson education India. Pearson Education India.

[99] Ohmer X, Marino M, Franke M, König P. 2022 Mutual influence between language and perception in multi-agent communication games. PLoS Comput. Biol. **18**, e1010658. (10.1371/journal.pcbi.1010658) 36315590 PMC9648844

[100] Tishby N, C.Pereira F, Bialek W. 1999 ‘The information bottleneck method’. Proceedings of the 37-th Annual Allerton Conference on Communication, Control and Computing 368–77. (10.48550/ARXIV.PHYSICS/0004057)

[101] Kirby S, Tamariz M, Cornish H, Smith K. 2015 Compression and communication in the cultural evolution of linguistic structure. Cognition **141**, 87–102. (10.1016/j.cognition.2015.03.016) 25966840

[102] Zaslavsky N, Kemp C, Regier T, Tishby N. 2018 Efficient compression in color naming and its evolution. Proc. Natl Acad. Sci. **115**, 7937–7942. (10.1073/pnas.1800521115) 30021851 PMC6077716

[103] Steels LL. 2015 The Talking Heads experiment: Origins of words and meanings. (Computational Models of Language Evolution 1). Berlin: Language Science Press. (10.26530/OAPEN_559870)

[104] Kirby S. 2001 Spontaneous evolution of linguistic structure-an iterated learning model of the emergence of regularity and irregularity. IEEE Trans. Evol. Comput. **5**, 102–110. (10.1109/4235.918430)

[105] Smith K, Kirby S, Brighton H. 2003 Iterated Learning: A Framework for the Emergence of Language. Artif. Life **9**, 371–386. (10.1162/106454603322694825) 14761257

[106] Vogt P. 2005 The emergence of compositional structures in perceptually grounded language games. Artif. Intell. **167**, 206–242. (10.1016/j.artint.2005.04.010)

[107] Perfors A, Navarro DJ. 2014 Language Evolution Can Be Shaped by the Structure of the World. Cogn. Sci. **38**, 775–793. (10.1111/cogs.12102) 24460933

[108] Christiansen MH, Chater N. 2008 Language as shaped by the brain. Behav. Brain Sci. **31**, 489–508; (10.1017/S0140525X08004998) 18826669

[109] Wellens P, Loetzsch M, Steels L. 2008 Flexible word meaning in embodied agents. Connect. Sci. **20**, 173–191. (10.1080/09540090802091966)

[110] Beuls K, Steels L. 2013 Agent-Based Models of Strategies for the Emergence and Evolution of Grammatical Agreement. PloS One (ed RV Solé), **8**, e58960. (10.1371/journal.pone.0058960) 23527055 PMC3601110

[111] Botoko Ekila J. 2024 Emergence of linguistic conventions in multi-agent systems through situated communicative interactions. In Proceedings of the 23rd International Conference on Autonomous Agents and Multiagent Systems, pp. 2725–2727. Richland, SC: International Foundation for Autonomous Agents and Multiagent Systems.

[112] Wagner K, Reggia JA, Uriagereka J, Wilkinson GS. 2003 Progress in the Simulation of Emergent Communication and Language. Adapt. Behav. **11**, 37–69. (10.1177/10597123030111003)

[113] Zhu C, Dastani M, Wang S. 2024 A survey of multi-agent deep reinforcement learning with communication. Auton. Agents Multi Agent Syst. **38**. (10.1007/s10458-023-09633-6)

[114] Sukhbaatar S, Szlam A, Fergus R. 2016 Learning multiagent communication with backpropagation. Adv. Neural Inf. Process. Syst. 2244–2252.

[115] Foerster JN, M.Assael Y, Freitas N, Whiteson S. 2016 ‘Learning to communicate with deep multi-agent reinforcement learning’. In Proceedings of the 30th International Conference on Neural Information Processing Systems, pp. 2145–2153. Red Hook, NY, USA: NIPS’16 Curran Associates Inc.

[116] Peng P, Wen Y, Yang Y, Yuan Q, Tang Z, Long H, Wang J. 2017 Multiagent Bidirectionally-Coordinated Nets: Emergence of Human-Level Coordination in Learning to Play StarCraft Combat Games. arXiv Preprint arXiv:1703.10069

[117] Wang Y, Sartoretti G. 2022 FCMNet: full communication memory net for team-level cooperation in multi-agent systems. In Proceedings of the 21st International Conference on Autonomous Agents and Multiagent System, pp. 1355–1363. Richland, SC: AAMAS. International Foundation for Autonomous Agents, Multiagent Systems. https://dl.acm.org/doi/abs/10.5555/3535850.3536001.

[118] Hoshen Y. 2017 ‘VAIN: attentional multi-agent predictive modeling.’Proceedings of the 31st. In International Conference on Neural Information Processing Systems, pp. 2698–2708. Red Hook, NY, USA: NIPS’17Curran Associates Inc. https://proceedings.neurips.cc/paper_files/paper/2017/hash/748ba69d3e8d1af87f84fee909eef339-Abstract.html.

[119] Jiang J, Lu Z. 2018 Learning attentional communication for multi-agent cooperation. In Advances in neural information processing systems (eds S Bengio, H Wallach, H Larochelle, K Grauman,, N Cesa-Bianchi, R Garnett), vol. 31. Curran, Associates, Inc.

[120] Abhishek D, Théophile G, Joshua R, Dhruv B, Devi P, Mike R, Joelle P. 2004 TarMAC: Targeted multi-agent communication. In Proceedings of the 36th International Conference on Machine Learning, vol. **97**, pp. 1538–1546, (10.1023/B:MACH.0000035472.73496.0c)

[121] Singh A, Jain T, Sukhbaatar S. 2019 ‘Learning when to communicate at scale in multiagent cooperative and competitive tasks. In International Conference on Learning Representations. https://openreview.net/forum?id=rye7knCqK7.

[122] Zhang SQ, Zhang Q, Lin J. 2019 Efficient communication in multi-agent reinforcement learning via variance based control. In Advances in neural information processing systems (eds H Wallach, H Larochelle, A Beygelzimer, F d’Alché-Buc, E Fox, R Garnett), vol. 32. Curran Associates, Inc.

[123] Wang R, He X, Yu R, Qiu W, An B, Rabinovich Z. 2004 Learning efficient multi-agent communication: An information bottleneck approach (eds H Daumé III, A Singh). In Proceedings of the 37th International Conference on Machine Learning, vol. **119**, pp. 9908–9918, (10.1023/b:mach.0000035472.73496.0c)

[124] Han S, Dastani M, Wang S. 2023 Model-based sparse communication in multi-agent reinforcement learning. In Proceedings of the 2023 International Conference on Autonomous Agents and Multiagent Systems, pp. 439–447. Richland, SC: International Foundation for Autonomous Agents and Multiagent Systems. https://dl.acm.org/doi/abs/10.5555/3545946.3598669.

[125] Cao K, Lazaridou A, Lanctot M, Z.Leibo J, Tuyls K, Clark S. 2018 Emergent communication through negotiation. In International Conference on Learning Representations. https://openreview.net/forum?id=Hk6WhagRW.

[126] Lazaridou A, M.Hermann K, Tuyls K, Clark S. 2018 ‘Emergence of linguistic communication from referential games with symbolic and pixel input. In International Conference on Learning Representations. https://openreview.net/forum?id=HJGv1Z-AW.

[127] Jaques N, Lazaridou A, Hughes E, Gulcehre C, A.Ortega P, Strouse D, Z.Leibo J, Freitas N. 2019 ‘Social influence as intrinsic motivation for multi-agent deep reinforcement learning’. In Proceedings of the 36th International Conference on Machine Learning, pp. 3040–3049.

[128] Kim D, Moon S, Hostallero D, J.Kang W, Lee T, Son K, Yi Y. 2019 Learning to schedule communication in multi-agent reinforcement learning. In International Conference on Learning Representations. https://openreview.net/forum?id=SJxu5iR9KQ.

[129] Rita M, Tallec C, Michel P, Grill JB, Pietquin O, Dupoux E, Strub F. 2022 Emergent communication: Generalization and overfitting in lewis games. In Advances in neural information processing systems (eds S Koyejo, S Mohamed, A Agarwal, D Belgrave, K Cho, A Oh), pp. 1389–1404, vol. **35**. Curran Associates, Inc.

[130] Mordatch I, Abbeel P. 2018 Emergence of Grounded Compositional Language in Multi-Agent Populations (eds SA McIlraith, KQ Weinberger). In Proceedings of the Thirty-Second AAAI Conference on Artificial Intelligence, vol. **32**, pp. 1495–1502, AAAI Press. (10.1609/aaai.v32i1.11492)

[131] Rita M, Strub F, Grill JB, Pietquin O, Dupoux E. 2022 ‘On the role of population heterogeneity emergent communication.’. In International Conference on Learning Representations. https://openreview.net/forum?id=5Qkd7-bZfI.

[132] Chaabouni R, Kharitonov E, Dupoux E, Baroni M. 2019 Anti-efficient encoding in emergent communication. In Advances in neural information processing systems (eds H Wallach, H Larochelle, A Beygelzimer, F d’Alché-Buc, E Fox, R Garnett), vol. 32. Curran Associates, Inc.

[133] Lowe R, Foerster J, Boureau YL, Pineau J, Dauphin Y. 2019 On the pitfalls of measuring emergent communication. In Proceedings of the 18th International Conference on Autonomous Agents and MultiAgent Systems, pp. 693–701. Richland, SC: AAMAS ’19.

[134] Lazaridou A, Baroni M. 2020 Emergent Multi-Agent Communication in the Deep Learning Era. arxiv https://arxiv.org/abs/2006.02419

[135] Bouchacourt D, Baroni M. 2018 How agents see things: On visual representations in an emergent language game. In Proceedings of the 2018 Conference on Empirical Methods in Natural Language Processing, pp. 981–985. Association for Computational Linguistics. (10.18653/v1/d18-1119)

[136] Harnad S. 1990 The symbol grounding problem. Phys. D **42**, 335–346. (10.1016/0167-2789(90)90087-6)

[137] Vogt P. 2002 The physical symbol grounding problem. Cogn. Syst. Res. **3**, 429–457. (10.1016/s1389-0417(02)00051-7)

[138] Lee J, Cho K, Weston J, Kiela D. 2018 Emergent translation in multi-agent communication. In International Conference on Learning Representations.

[139] Lin T, Huh M, Stauffer C, Lim SN, Isola P. 2021 Learning to ground multi-agent communication with autoencoders. In Advances in Neural Information Processing Systems.

[140] Das A, Kottur S, Moura JMF, Lee S, Batra D. 2017 Learning Cooperative Visual Dialog Agents with Deep Reinforcement Learning. In Proceedings of the IEEE International Conference on Computer Vision (ICCV).

[141] Havrylov S, Titov I. 2017 Emergence of language with multi-agent games: learning to communicate with sequences of symbols. In Proceedings of the 31st International Conference on Neural Information Processing Systems, pp. 2146–2156. Red Hook, NY, USA: NIPS’17 Curran Associates Inc.

[142] Gupta A, Lanctot M, Lazaridou A, P.Liang. 2021 Dynamic population-based meta-learning for multi-agent communication with natural language (eds Y A.Beygelzimer, J WortmanVaughan). In Advances in Neural Information Processing Systems.

[143] Tucker M, Li H, Agrawal S, Hughes D, P.Sycara K, Lewis M, Shah J, P.Liang. 2021 (eds Y A.Beygelzimer, J WortmanVaughan). In Advances in Neural Information Processing Systems.

[144] Lee J, Cho K, Kiela D. 2019 Countering Language Drift via Visual Grounding. In Proceedings of the 2019 Conference on Empirical Methods in Natural Language Processing and the 9th International Joint Conference on Natural Language Processing (EMNLP-IJCNLP), pp. 4385–4395. Hong Kong, China: Association for Computational Linguistics.

[145] Lazaridou A, Potapenko A, Tieleman O. 2020 Multi-agent Communication meets Natural Language: Synergies between Functional and Structural Language Learning. In Proceedings of the 58th annual meeting of the association for computational linguistics, pp. 7663–7674. Association for Computational Linguistics.

[146] Lowe R, Gupta A, Foerster J, Kiela D, Pineau J. 2020 ‘On the interaction between supervision and self-play emergent communication.’. In International Conference on Learning Representations. https://openreview.net/forum?id=rJxGLlBtwH.

[147] Lazaridou A, Peysakhovich A, Baroni M. 2017 Multi-agent cooperation and the emergence of (natural) language. In International Conference on Learning Representations.

[148] Karten S, Tucker M, Kailas S, Sycara K. 2023 Towards True Lossless Sparse Communication in Multi-Agent Systems. In International Conference on Robotics and Automation (ICRA). (10.1109/icra48891.2023.10161322)

[149] Driess D, Xia F, Sajjadi MSM, Lynch C, Chowdhery A, Ichter B, Wahid A. 2023 Palm-e: An embodied multimodal language model. In Proceedings of the 40th International Conference on Machine Learning. vol. 202. PMLR.

[150] Carta T, Romac C, Wolf T, Lamprier S, Sigaud O, Oudeyer PY. 2023 Grounding Large Language Models in Interactive Environments with Online Reinforcement Learning. In Proceedings of Machine Learning Research, vol. **202**, pp. 3676–3713, PMLR.

[151] Christiano PF, Leike J, Brown T, Martic M, Legg S, Amodei D. 2017 Deep reinforcement learning from human preferences (eds I Guyon, U Von Luxburg, S Bengio, H Wallach, R Fergus, S Vishwanathan, R Garnett). In Advances in Neural Information Processing Systems. Curran Associates, Inc.

[152] Zhu F, Simmons R. 2024 Bootstrapping Cognitive Agents with a Large Language Model. Proceedings of the AAAI Conference on Artificial Intelligence **38**, 655–663. (10.1609/aaai.v38i1.27822)

[153] Zhang H, Du W, Shan J, Zhou Q, Du Y, B.Tenenbaum J, Shu T, Gan C. 2024 ‘Building cooperative embodied agents modularly with large language. In International Conference on Learning Representations.

[154] Wu Q, Bansal G, Zhang J, Wu Y, Li B, Zhu E, Jiang L. 2023 AutoGen: Enabling next-Gen LLM Applications via Multi-Agent Conversation. arXiv preprint arXiv:2308.08155.

[155] Li G, Hammoud H, Itani H, Khizbullin D, Ghanem B. 2023 CAMEL: communicative agents for ‘Mind’ exploration of large language model society. Adv. Neural Inf. Process. Syst. (eds A Oh, T Neumann, A Globerson, K Saenko, M Hardt, S Levine), **36**, 51991–52008.

[156] Park JS, O’Brien J, Cai CJ, Morris MR, Liang P, Bernstein MS. 2023 Generative Agents: Interactive Simulacra of Human Behavior. In Proceedings of the 36th Annual ACM Symposium on User Interface Software and Technology. UIST ’23 Association for Computing Machinery.

[157] Vezhnevets AS *et al*. 2023 Generative Agent-Based Modeling with Actions Grounded in Physical, Social, or Digital Space Using Concordia. arXiv Preprint arXiv:2312.03664

[158] Perez J, Léger C, Ovando-Tellez M, Foulon C, Dussauld J, Oudeyer PY, Moulin-Frier C. 2024 Cultural Evolution in Populations of Large Language Models. arXiv Preprint arXiv:2403.08882

[159] Wei J, Wang X, Schuurmans D, Bosma M, brianichter FX, Chi E, V.Le Q, Zhou D. 2022 Chain-of-thought prompting elicits reasoning in large language models. In Advances neural information processing systems (eds S Koyejo, S Mohamed, A Agarwal, D Belgrave, K Cho, A Oh), pp. 24824–24837, vol. 35. Curran Associates, Inc. See https://proceedings.neurips.cc/paper_files/paper/2022/file/9d5609613524ecf4f15af0f7b31abca4-Paper-Conference.pdf.

[160] Liu J, Yu C, Gao J, Xie Y, Liao Q, Wu Y, Wang Y. 2024 ‘LLM-powered hierarchical language agent for real-time human-AI coordination’. In International Conference on Autonomous Agents and Multiagent Systems. https://www.ifaamas.org/Proceedings/aamas2024/pdfs/p1219.pdf.

[161] Hunt W, Godfrey T, D.Soorati M. 2024 ‘Conversational language models for human-in-the-loop multi-robot coordination’. In Demonstration at International Conference on Autonomous Agents and Multi-Agent Systems. https://www.ifaamas.org/Proceedings/aamas2024/pdfs/p2809.pdf.

[162] Acerbi A, Stubbersfield JM. 2023 Large language models show human-like content biases in transmission chain experiments. Proc. Natl Acad. Sci. USA. **120**, e2313790120. (10.1073/pnas.2313790120) 37883432 PMC10622889

[163] Ji Z *et al*. 2023 Survey of Hallucination in Natural Language Generation. ACM Comput. Surv. **55**, 1–38. (10.1145/3571730)

[164] Fudenberg D, Levine D. 1998 Learning in games. Eur. Econ. Rev. **42**, 631–639. (10.1016/s0014-2921(98)00011-7)

[165] Nowak MA. 2006 Evolutionary dynamics: exploring the equations of life. Harvard university press. (10.2307/j.ctvjghw98)

[166] Sen P, K.Chakrabarti B. 2014 Sociophysics: an introduction. OUP Oxford.

[167] Galam S, Gefen (Feigenblat) Y, Shapir Y. 1982 Sociophysics: A new approach of sociological collective behaviour. I. mean‐behaviour description of a strike. J. Math. Sociol. **9**, 1–13. (10.1080/0022250x.1982.9989929)

[168] Tailleur J, Gompper G, C.Marchetti M, M.Yeomans J, Salomon C. 2022 Active Matter and Non-equilibrium Statistical Physics. Lecture notes of the les houches summer school, vol. 112. Oxford University Press. (10.1093/oso/9780192858313.001.0001). See https://academic.oup.com/book/45056.

[169] Eiben AE, Smith JE. 2015 Evolutionary Computing: The Origins. In Natural computing series introduction to evolutionary computing, pp. 13–24. Springer. (10.1007/978-3-662-44874-8_2)

[170] Sutton RS. 2018 Reinforcement learning: An Introduction. In A bradford book. (10.1007/978-1-4615-3618-5_1)

